# In Vivo Characterization of a Novel **γ**-Secretase Inhibitor SCH 697466 in Rodents and Investigation of Strategies for Managing Notch-Related Side Effects

**DOI:** 10.1155/2013/823528

**Published:** 2013-03-14

**Authors:** Lynn A. Hyde, Qi Zhang, Robert A. Del Vecchio, Prescott T. Leach, Mary E. Cohen-Williams, Lei Chen, Gwendolyn T. Wong, Nansie A. McHugh, Joseph Chen, Guy A. Higgins, Theodros Asberom, Wei Li, Dmitri Pissarnitski, Diane Levitan, Amin A. Nomeir, John W. Clader, Lili Zhang, Eric M. Parker

**Affiliations:** ^1^Department of Neuroscience, Merck Research Laboratories, Kenilworth, NJ 07033, USA; ^2^Department of Molecular Biomarkers, Merck Research Laboratories, Kenilworth, NJ 07033, USA; ^3^Department of Medicinal Chemistry, Merck Research Laboratories, Kenilworth, NJ 07033, USA; ^4^Department of Drug Metabolism and Pharmoackinetics, Merck Research Laboratories, Kenilworth, NJ 07033, USA

## Abstract

Substantial evidence implicates *β*-amyloid (A*β*) peptides in the etiology of Alzheimer's disease (AD). A*β* is produced by the proteolytic cleavage of the amyloid precursor protein by *β*- and *γ*-secretase suggesting that *γ*-secretase inhibition may provide therapeutic benefit for AD. Although many *γ*-secretase inhibitors have been shown to be potent at lowering A*β*, some have also been shown to have side effects following repeated administration. All of these side effects can be attributed to altered Notch signaling, another *γ*-secretase substrate. Here we describe the in vivo characterization of the novel *γ*-secretase inhibitor SCH 697466 in rodents. Although SCH 697466 was effective at lowering A*β*, Notch-related side effects in the intestine and thymus were observed following subchronic administration at doses that provided sustained and complete lowering of A*β*. However, additional studies revealed that both partial but sustained lowering of A*β*and complete but less sustained lowering of A*β* were successful approaches for managing Notch-related side effects. Further, changes in several Notch-related biomarkers paralleled the side effect observations. Taken together, these studies demonstrated that, by carefully varying the extent and duration of A*β* lowering by *γ*-secretase inhibitors, it is possible to obtain robust and sustained lowering of A*β* without evidence of Notch-related side effects.

## 1. Introduction

Alzheimer's disease (AD) is a progressive age-related neurodegenerative disease characterized clinically by memory loss and cognitive dysfunction followed by a disruption of normal daily functions, organ system failure, and, ultimately, death. However, a diagnosis of AD can only be confirmed postmortem by the presence of distinct neuroanatomical hallmarks including senile plaques consisting primarily of *β*-amyloid (A*β*) peptides, neurofibrillary tangles consisting of hyperphosphorylated tau, and substantial neuronal loss, particularly in the hippocampus, an area of the brain which plays a key role in memory.

Substantial genetic and neuroanatomical evidence implicates A*β* peptides in the etiology of Alzheimer's disease (e.g., [1–3]). Therefore, it is thought that a reduction in A*β* production or an increase in A*β* clearance will have a beneficial, and potentially disease modifying, effect on the disease. 

A*β* is produced by sequential cleavage of amyloid precursor protein (APP) by *β*-site APP cleaving enzyme 1 (BACE1) followed by *γ*-secretase. Thus, inhibiting *γ*-secretase should decrease A*β* production. Indeed, acute and chronic administration of small molecule *γ*-secretase inhibitors reduced A*β* in the plasma, brain and cerebrospinal fluid (CSF) of animals [4–12] and humans [8, 13–15], including AD patients [16, 17]. More importantly, *γ*-secretase inhibition has also been reported to slow and even halt the progression of amyloid plaque deposition in mouse models of amyloid production and deposition [18, 19]. 

However, the C-terminal fragment of APP (the product of BACE1 cleavage) is not the only substrate for *γ*-secretase [20, 21]. Additional physiologically relevant substrates include Notch [22], which has been shown to play a critical role in cell fate pathways [23]. Thus, *γ*-secretase inhibitors also disrupt Notch signaling. Although some “Notch sparing” (e.g., [8, 24–26]) and “APP selective” [10] *γ*-secretase inhibitors have been identified and characterized, other *γ*-secretase inhibitors have been shown to produce mechanism-based, Notch-related side effects following chronic administration in vivo including a reduction in thymus weight and intestinal goblet cell hyperplasia [5, 27–29]. These effects are usually observed at doses that provide sustained and near complete lowering of A*β*, in particular in the periphery (i.e., plasma A*β*).

Since there is a risk of Notch-related side effects with chronic administration of *γ*-secretase inhibitors, methods of minimizing or completely avoiding these side effects are highly desirable. Identifying relevant Notch-related biomarkers could be a useful means of managing Notch-related side effects in clinical trials involving *γ*-secretase inhibitors. HES-1 is a direct target of the Notch signaling pathway, while KLF4, mATH-1, and adipsin are downstream targets of HES-1 expression [30–33]. It would be of interest to determine to what extent changes in gene expression of these downstream targets of Notch correlate with Notch-related pathology in vivo following subchronic inhibition of *γ*-secretase. In addition, it has been shown that partial inhibition of *γ*-secretase is an effective way to reduce A*β* with no evidence of Notch-related side effects in rodents [5, 18]. However, it is not clear if partial A*β* lowering and/or complete A*β* lowering for a part of the day are effective methods for minimizing Notch-related side effects.

Here we describe the in vivo characterization of a novel *γ*-secretase inhibitor, SCH 697466 [34]. We have assessed A*β* lowering in rats, transgenic CRND8 mice (TgCRND8, mouse model of amyloid production and deposition [35]) and nontransgenic mice following acute and subchronic administration. Using SCH 697466 as a tool, we have also attempted to identify strategies to maintain A*β* lowering while managing Notch-related side effects in vivo by varying the extent and duration of A*β* lowering. Further, we measured gene expression of several downstream targets of Notch in the intestine and blood as potential safety biomarkers for Notch disruption.

## 2. Materials and Methods

### 2.1. Animals

Male CD rats (115–175 g; Crl:CD(SD); Charles River Laboratories, Kingston, NY) were group housed, and female nontransgenic B6C3F1 mice (6 weeks; Charles River Laboratories, Raleigh, NC; the background strain on which TgCRND8 mice are maintained) were single housed; all groups were acclimated to the vivarium for one week prior to use in a study. 

The transgenic (Tg) CRND8 male and female (counterbalanced across groups) mice (carrying the Swedish and Indiana familial Alzheimer's disease APP mutations under the control of the Syrian hamster prion protein [35]) used in these studies were bred at Merck Research Laboratories in Kenilworth, NJ, or Taconic in Germantown, NY, as described previously [36]. Before dosing began and for the duration of the study, mice were singly housed with a plastic igloo and nesting material. TgCRND8 mice were 5–7 weeks old (preplaque) because at this age total cortical A*β* is primarily in a soluble form [36] and thus is more amenable to reduction following relatively short-term *γ*-secretase inhibition [37, 38] (unpublished observations show better survival if TgCRND8 mice are single housed versus group housed).

Table 1 describes the various studies conducted in rats, TgCRND8 mice, and nontransgenic mice. Body weight was assessed prior to drug administration. All in vivo procedures adhered to the NIH Guide for the Care and Use of Laboratory Animals and were approved by the Institutional Animal Care and Use Committee of Merck Research Laboratories in Kenilworth, an AAALAC accredited institution.

### 2.2. SCH 697466

**Figure 1:**
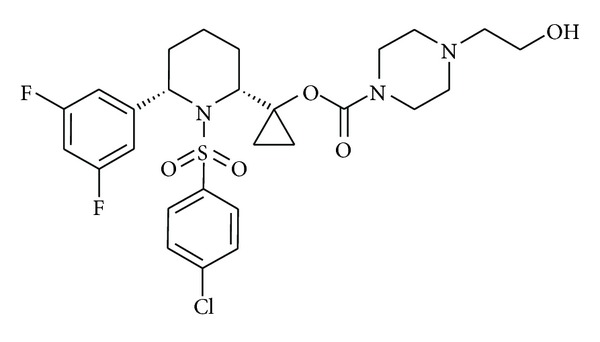
SCH 697466: 1-[cis-1-[(4-chlorophenyl)sulfonyl]-6-(3,5-difluorophenyl)-2-piperidinyl]cyclopropyl 4-(2-hydroxyethyl)-1-piperazinecarboxylate [34].

SCH 697466 (Figure 1) is a novel sulfonamide *γ*-secretase inhibitor [34]. It was synthesized by the Medicinal Chemistry group at Merck Research Laboratories in Kenilworth, NJ, and formulated in 20% hydroxypropyl-*β*-cyclodextrin for all studies, except for the 6-day TgCRND8 study where the vehicle was diluted 1 : 10 with 0.4% methylcellulose. SCH 697466 was administered orally to both mice and rats, and the dosing volume was 10 mL/kg for mice and 5 mL/kg for rats. 

### 2.3. *β*-Amyloid Quantification

In vitro procedures for measuring *γ*-secretase activity in membranes prepared from HEK293 cells expressing APP and procedures for collecting tissues and assessing A*β*40 levels in plasma and cortex (following guanidine extraction; biotin-4G8 and S-tag G2-10 antibodies for transgenic and nontransgenic mice and antibody 585 and S-tag G2-10 for rats) have been previously described [28, 39]. Plasma and cortex A*β*42 were quantified in select TgCRND8 studies using biotin-4G8 and S-tag G2-11 and 4G8 antibodies.

Cerebrospinal fluid (CSF) was collected from the cisterna magna immediately following euthanasia with excess CO_2_ and quickly frozen on dry ice and stored at −70°C until A*β* quantification using procedures identical to those used to quantify plasma A*β*. Only visibly clear CSF samples were analyzed. CSF was not collected in some studies because the studies were performed before technique was developed and optimized.

### 2.4. Histology

The thymus was weighed as previously described [5]. The ileum (harvested just proximal to the ileocecal junction) was processed, sectioned (3 *μ*m), and stained as previously described [28], and intestinal goblet cell hyperplasia was quantified as percent villi area covered by Periodic Acid-Schiff (PAS) stain as previously described [5].

### 2.5. Notch Pathway Biomarker Analyses

The jejunum was harvested, rinsed, and flash frozen in liquid nitrogen until later analyses. RNA was isolated from tissues using the Trizol reagent (Molecular Research Center, Inc.) followed by purification over an RNAeasy column (Qiagen).

White blood cells were isolated after collection in heparin tubes. Tubes were centrifuged for 10 minutes at 5000 rpm, and the interphase cells were removed to a tube containing Trizol reagent. RNA was purified over an RNAeasy column. Quantitative, real-time PCR was performed on an ABI7900 machine (Applied Biosystems, Foster City, CA), using the Bio-Rad iScript Custom one-step RT-PCR Kit for Probes with ROX (Hercules, CA). Primer and probe were designed using the Universal Probe Library Assay Design Center (Roche Applied Sciences, Basel, Switzerland). The probes and primers used are as follows:

 KLF4: Forward primer CGGGAGGGAGAAGACACT; Reverse primer CGTTGGCGTGAGGGAAC PROBE#62 from Roche Universal Probe Library;
 HES1: Forward primer ATCCCGGTCTACACCAGCAA; Reverse primer AAGGTCACCGAGGAG PROBE#20 from Roche Universal Probe Library;
 Adipsin: Forward primer CTGGGAGCGGCTGTATGT; Reverse primer TCCTACATGGCTTCCGTG PROBE#79 from Roche Universal Probe Library;
 mATH1: Forward primer TCCCCTTCCTCCTACCTTCT; Reverse primer TGTACCTTTACGTGGCATCG PROBE#34 from Roche Universal Probe Library.

### 2.6. Quantification of Drug Levels

Plasma and brain concentrations of SCH 697466 were quantified by liquid chromatography tandem mass spectrometry (LC-MS/MS) using an Acquity UPLC HSS T3 C18 column (2.1 × 50 mm, 1.7 *μ*), operated at 50°C. The flow rate was 0.75 mL/min, and the instrument used was a Sciex API 4000/5500 mass spectrometer equipped with a Turbo Ion Spray source and operated at unit mass resolution. Plasma and brain samples were quantified against standard curves generated in the corresponding matrix. Standard curves for mouse plasma were generated from frozen heparinized plasma spiked with SCH 697466 to give final concentrations in the range of 1 ng/mL—5000 ng/mL. In the case of the mouse brains, control and sample brains were placed in a 48-well plate and 3 mL of water was added for each gram of tissue. Glass beads were added into each well, and samples were homogenized using an SPEX SamplePrep 2010 apparatus for 2.5 min at 1450 rpm. The calibration standards for brain were prepared from 1 ng/g to 10,000 ng/g. Brain homogenate and plasma samples and standards were prepared for analysis in the same manner, by subjecting them to protein precipitation with acetonitrile containing internal standard. The samples were vortexed and centrifuged and the supernatants were transferred to a 96-well plate for LC-MS/MS analysis.

### 2.7. Data Analyses

Analyses of variance (ANOVA) with dose group as the between-subject factor followed by Dunnett post hoc tests comparing the vehicle-treated group to all others were used to analyze most data. 

During subchronic administration of 100 mg/kg of SCH 697466 to TgCRND8 mice, all mice showed signs of poor health (e.g., hypothermic, hypoactive, poorly groomed, and hunched posture) and some died. If a mouse died prior to the tissue collection day, no tissues were used. If an animal died on the tissue collection day, thymus and ileum were isolated. If an animal was in very poor health on the collection day, it was not possible to obtain enough blood for A*β* quantification or exposure, but all other tissues were used. 

## 3. Results

### 3.1. SCH 697466 Is a Potent *γ*-Secretase Inhibitor In Vitro

SCH 697466 is a potent *γ*-secretase inhibitor when assessed in a variety of assay formats. The IC_50_ for A*β*40 inhibition in membranes prepared from human embryonic kidney 293 (HEK293) cells expressing human APP with the Swedish and London familial AD mutations (APP^Swe-Lon^) is 2 nM. In intact HEK293 cells expressing human APP^Swe-Lon^, SCH 697466 inhibits A*β*40 and A*β*42 production with IC_50_s of 5 and 3 nM, respectively. SCH 697466 also inhibits Notch processing in intact HEK293 cells expressing a portion of the human Notch1 protein with an IC_50_ of 123 nM. Thus, in vitro, there is a 24-fold separation between A*β*40 inhibition and Notch.

### 3.2. SCH 697466 Dose-Dependently Reduced A*β* in Plasma, Cortex, and/or Cerebrospinal Fluid in Rats and TgCRND8 and Nontransgenic Mice following Acute Administration

**Figure 2:**
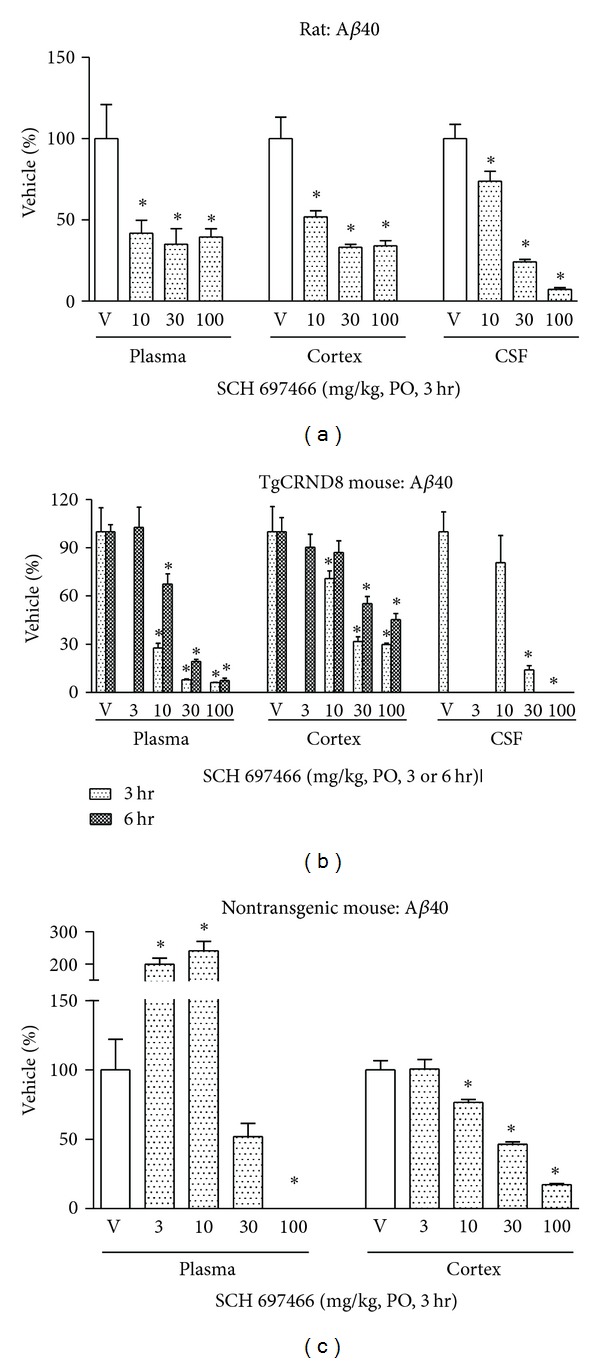
A*β*40 levels expressed as a percent of vehicle in plasma, cortex, and CSF following acute administration of SCH 697466 to (a) rats, (b) TgCRND8 mice, and (c) nontransgenic B6C3F1 mice. For the TgCRND8 studies (b) CSF was not collected in the 6 hr study and 3 mg/kg SCH 697466 was not included in the 3 hr study. *P < 0.02 versus vehicle, Dunnett post hoc test.

In rats, 3 hr after administration of SCH 697466, plasma, cortex, and CSF A*β*40 levels were significantly reduced (Figure 2(a); main effect of dose: *F*(3,26) = 6.37 and *F*(3,28) = 19.44 and 64.86, *P* < 0.003, for plasma, cortex, and CSF, resp.) with significant A*β* lowering in each compartment at all doses (*P* < 0.006). The effect of SCH 697466 on A*β* seemed to plateau at about 60% reduction in both plasma and cortex, while near complete lowering of A*β* was observed in CSF at the 100 mg/kg dose. The inclusion of lower doses may have provided a better understanding of the dose-response relationship of this compound in rats. 

In TgCRND8 mice, 3 hr after-drug administration, SCH 697466 dose-dependently reduced A*β*40 levels in the plasma, cortex, and CSF (Figure 2(b); *F*(3,13) = 36.35 and 17.17 and *F*(3,10) = 12.97, resp., *P* < 0.0009) with significant lowering at all doses in each compartment (*P* < 0.006, except 10 mg/kg in CSF). Similar to what was observed at 3 hr, six hr after administration, SCH 697466 continued to produce a dose-dependent reduction of plasma and cortex A*β*40 in TgCRND8 mice (Figure 2(b); main effect of dose: *F*(4,22) = 41.86 and 12.52, resp., *P* < 0.0001) with near complete lowering of plasma A*β*40 at 100 mg/kg. Six hr after-administration, SCH 697466 was similarly effective at lowering A*β*42 in plasma and brain such that the 100 mg/kg dose reduced plasma and cortex A*β*42 by 84% and 47%, respectively (data not shown), compared to 93% and 55% for plasma and cortex A*β*40. SCH 697466 seemed to be more potent in plasma and cortex 3 hr following administration compared to 6 hr, although the 3 hr and 6 hr studies were not conducted or analyzed together.

In nontransgenic mice, 3 hr after administration, SCH 697466 increased plasma A*β* at lower doses while it decreased A*β* at higher doses (Figure 2(c); main effect of dose: *F*(4,30) = 30.79, *P* < 0.0001); significant increase in A*β* was observed at 3 and 10 mg/kg and significant lowering of A*β* was observed at 100 mg/kg (*P* < 0.005). In the cortex, SCH 697466 dose-dependently reduced A*β* [main effect of dose: *F*(4,30) = 63.23, *P* < 0.0001) with significant lowering for the 10, 30, and 100 mg/kg groups (*P* < 0.005). Near complete lowering of A*β* was observed in plasma and cortex at 100 mg/kg.

There was a dose related increase (although in some cases it was more than dose-proportional) in the concentration of SCH 697466 in plasma and brain (one time point per animal), and the concentrations were usually greater in plasma than brain in rats, TgCRND8 mice, and nontransgenic mice (Table 2). The *T*_max⁡_ of SCH 697466 in rats is 2 hr (from a separate pharmacokinetic study) which basically agrees with our results in TgCRND8 mice showing that plasma concentrations were higher at 3 hr after administration than 6 hr. The concentration of SCH 697466 in plasma and brain tended to be higher in TgCRND8 mice compared to nontransgenic mice, but these studies were not conducted together. Nevertheless, there are no major species differences in the concentration of SCH 697466 in plasma and brain when comparing rats to TgCRND8 and nontransgenic mice.

### 3.3. Duration of A*β* Lowering in Plasma and Cortex following a Single Dose of SCH 697466 in TgCRND8 Mice

To determine the duration of action of SCH 697466 on lowering plasma and cortex A*β* over 24 hr, two time course studies were conducted.

**Figure 3:**
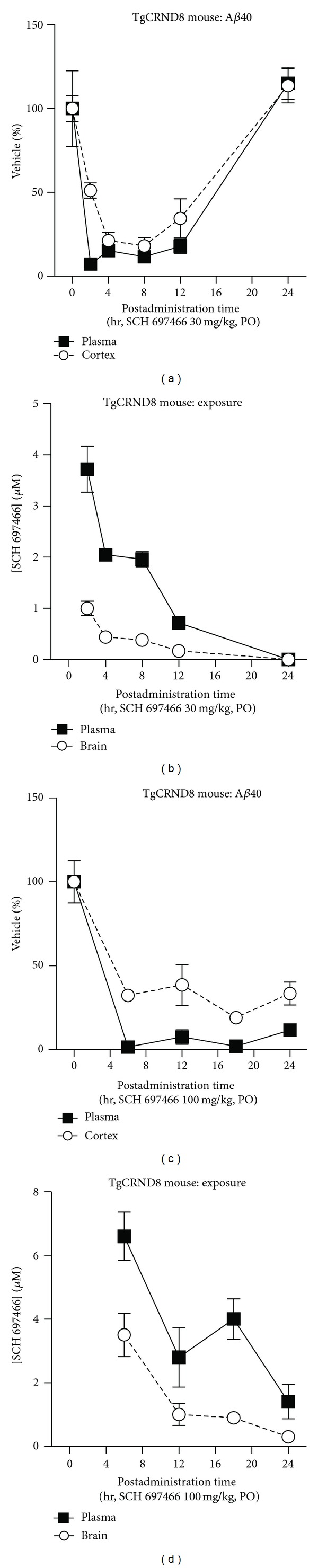
A*β*40 levels over time expressed as a percent of vehicle in plasma and cortex following a single dose of (a) 30 mg/kg or (c) 100 mg/kg of SCH 697466 in TgCRND8 mice. Plasma and brain concentration (*μ*M) of SCH 697466 following a single dose of (b) 30 mg/kg or (d) 100 mg/kg of SCH 697466 in TgCRND8 mice.

Following a single dose of 30 mg/kg of SCH 697466 in TgCRND8 mice, plasma and cortex A*β*40 levels were significantly reduced at 2, 4, 8, and 12 hr (Figure 3(a); main effect of time point: *F*(5,23) = 85.76 and 15.02, *P* < 0.0001; Dunnett test: *P* < 0.02); however, 24 hr after dosing, A*β* levels had returned to baseline and were not different from vehicle (*P* > 0.20). SCH 697466 was not quite as potent at lowering plasma and cortex A*β*42 compared to A*β*40 (data not show); for example, 8 hr after-dose, SCH 697466 lowered plasma and cortex A*β*42 by 80% and 42%, respectively. compared to 89% and 82% for plasma and cortex A*β*40. There was a time-dependent reduction in the concentration of SCH 697466 in plasma and brain with very little remaining 24 hr later, respectively (Figure 3(b)). 

A single dose of 100 mg/kg of SCH 697466 inhibited A*β* in the plasma and cortex 6, 12, 18, and 24 hr after administration (Figure 3(c); main effect of time point: *F*(4,20) = 46.95 and 20.72, *P* < 0.0001; Dunnett test: *P* < 0.0001). There was a time-dependent reduction in the concentration of SCH 697466 in plasma and brain with 1.4 and 0.3 *μ*M remaining 24 hr later (Figure 3(d)).

### 3.4. SCH 697466 Reduced Plasma and Cortex A*β*, but Produced Notch-Related Side Effects following Subchronic Administration in TgCRND8 Mice

As has been reported previously [5, 27–29], upon repeated administration some *γ*-secretase inhibitors produce mechanism-based Notch-related side effects including a reduction in thymus weight and an increase in goblet cell hyperplasia in the intestine. In an effort to determine the potential Notch-related side effect liability and the therapeutic window of SCH 697466 in rodents, we tested SCH 697466 in our TgCRND8 rapid 6-day therapeutic index assay [5]. In this assay, we include doses that provide continuous, near complete lowering A*β* in plasma. Since 30 mg/kg of SCH 697466 provided reasonable lowering (~80% reduction) of A*β* in plasma for 12 hr after administration, but not 24 hr, we decided to administer all doses of SCH 697466 *b.i.d.*

#### 3.4.1. A*β* Lowering

**Figure 4:**
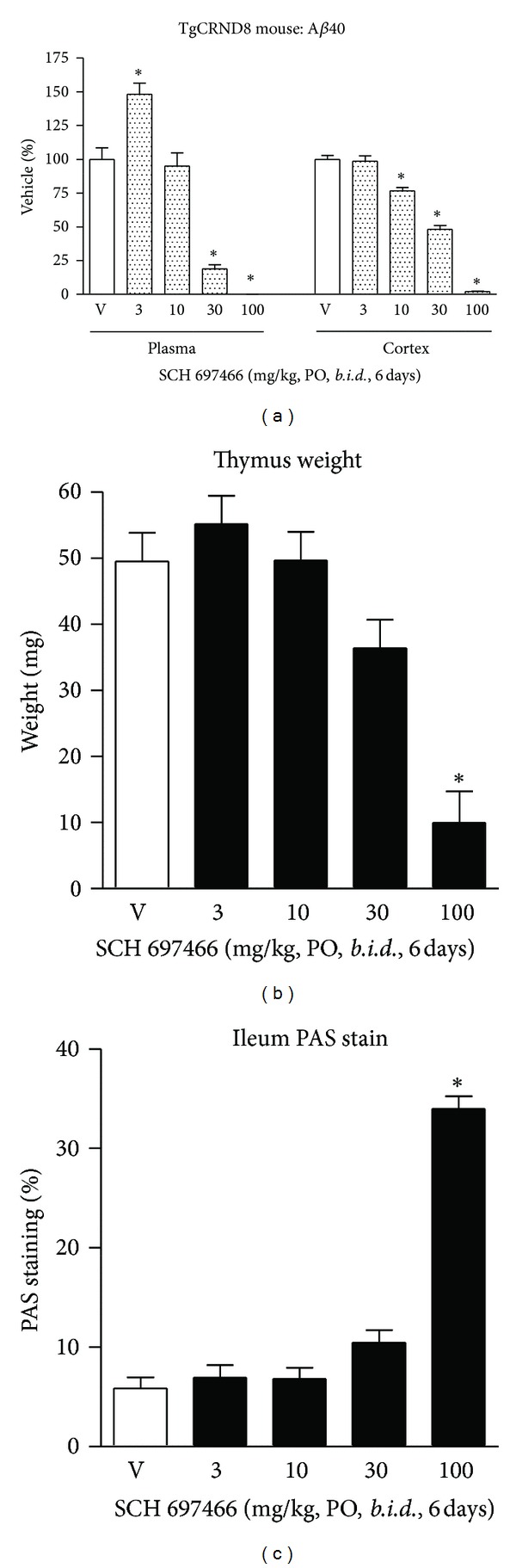
(a) A*β*40 levels expressed as a percent of vehicle in plasma and cortex, (b) thymus weight, and (c) percent area in the ileum covered by PAS stain following 6 days of *b.i.d.* administration of SCH 697466 to TgCRND8 mice. The final dose of SCH 697466 was given on day six 3 hr prior to tissue harvesting. *P < 0.0006 versus vehicle, Dunnett post hoc test.

Six days of *b.i.d.* dosing with SCH 697466 in TgCRND8 mice (5 full days with a final dose 3 hr prior to sacrifice on day 6) significantly reduced plasma and cortical A*β*40 levels (Figure 4(a); main effect of dose: *F*(4,29) = 49.76 and *F*(4,30) = 106.92, *P* < 0.0001, for plasma and cortex, resp.) with near complete lowering of in both compartments at 100 mg/kg. There was a significant increase of A*β*40 in plasma with the 3 mg/kg dose. SCH 697466 was not as potent at lowering A*β*42 in plasma and brain compared to A*β*40, such that the 100 mg/kg dose reduced plasma and cortex A*β*42 by 90% and 66%, respectively (data not shown), compared to 100% and 98% for plasma and cortex A*β*40. 

#### 3.4.2. Notch-Related Side Effects

Six days of *b.i.d.* administration of SCH 697466 significantly reduced thymus weight (Figure 4(b); main effect of dose: *F*(4,19) = 15.59, *P* < 0.002) with a significant reduction observed in the 100 mg/kg dose group (*P* < 0.003), but not the 30 mg/kg group. 

This dosing regime also increased PAS staining in the ileum (Figure 4(c); main effect of dose: *F*(4,17) = 91.34, *P* < 0.0001) such that there was almost a 6-fold increase in the 100 mg/kg group compared to vehicle treated mice (Figures 4(d) and 4(e); *P* < 0.0001), but not the 30 mg/kg group (just under a 2-fold increase). 

Further, mice treated with 100 mg/kg of SCH 697466 steadily lost weight during the 6 days of drug administration; 100 mg/kg mice lost 4.3 g over 6 days while vehicle-treated mice lost only 1 g over the same time period and 66.6% of the mice in the 100 mg/kg group died before the study was completed (compared to 12.5% in the vehicle treated group), and the surviving mice appeared in poor health by the last day of the study (e.g., hypothermic, hunched posture, etc.).

#### 3.4.3. Exposure

There was a dose-related increase (although in some cases it was more than dose proportional) in the concentration of SCH 697466 in plasma and brain (one time point per animal) (Table 3). Drug concentrations following 6 days of *b.i.d.* administration were similar to those observed following a single dose (3 hr) with the exception of the 100 mg/kg group where there was much greater brain exposure following 6 days of *b.i.d.* dosing than following a single dose (c.f., Tables 2 and 3). Due to the poor condition of the mice, there was insufficient plasma to obtain exposure data for mice in the 100 mg/kg group.

### 3.5. Two Different Dosing Paradigms of SCH 697466 in Nontransgenic Mice Provided Equivalent Lowering of Plasma and Cortex A*β* but Resulted in Different Observations of Notch-Related Side Effects and Biomarker Changes

The time course study suggested that both *q.d.* and *b.i.d.* administration of SCH 697466 at 100 mg/kg would provide sustained and near complete lowering of A*β*, and the subchronic study suggested that Notch-related side effects would be observed under conditions of sustained and near complete lowering of A*β*. In an effort to determine whether Notch-related side effects could be managed by giving SCH 697466 *q.d.* instead of *b.i.d.*, mice were orally administered SCH 697466 either once a day for 11 days (11 days *q.d.*) or twice a day for 5 days with the final dose on the 6th day (6 days *b.i.d.*) with both groups being given a total of 11 doses. All tissues were collected 3-4 hr after the last dose. In addition, we assessed changes in expression of genes downstream of Notch in the intestine and blood to assess the potential of these measures to be useful biomarkers for Notch-related pathology. This study was conducted in nontransgenic B6C3F1 mice since as noted above TgCRND8 mice did not tolerate high doses of SCH 697466.

#### 3.5.1. A*β* Lowering

**Figure 5:**
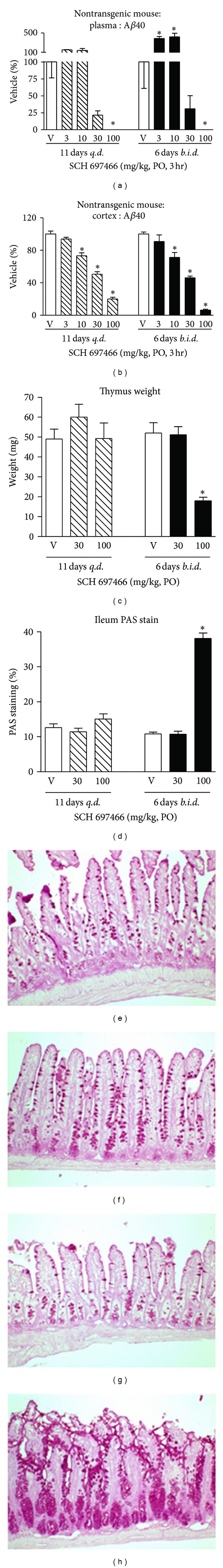
A*β*40 levels expressed as a percent of vehicle in (a) plasma and (b) cortex, (c) thymus weight, and (d) percent area in the ileum covered by PAS stain following 11 days of *q.d.* administration (left side of graphs) or 6 days of *b.i.d.* administration (right side of graphs) of SCH 697466 in nontransgenic B6C3F1 mice. The final dose of SCH 697466 was given 3 hr prior to tissue harvesting. Representative examples of the PAS stained ileum from (e) 11 day *q.d.* vehicle-treated mouse; 13% PAS staining, (f) 11 day *q.d.* 100 mg/kg SCH 697466 treated mouse; 16% PAS staining, (g) 6 day *b.i.d.* vehicle-treated mouse; 11% PAS staining, and (h) 6 day *b.i.d.* 100 mg/kg SCH 697466 treated mouse; 39% PAS staining. Increased PAS staining was only observed in the 6 day *b.i.d. *100 mg/kg treated group. *P < 0.003 versus vehicle, Dunnett post hoc test.

For each dosing paradigm, the effects of SCH 697466 on A*β* in the plasma of nontransgenic mice were nonlinear such that lower doses increased A*β*40 levels while higher doses decreased A*β*40 levels (Figure 5(a); main effect of dose: *F*(4,19) = 11.02 and *F*(4,21) = 38.43, *P* < 0.0001 for 11 days *q.d.* and 6 days *b.i.d.* groups, resp.). The effects of SCH 697466 on A*β* in the plasma were similar between the dosing paradigms with 50% lowering of A*β* occurring at a dose between 10 and 30 mg/kg and complete lowering of A*β* at 100 mg/kg. Plasma A*β* lowering was similar to what was observed in TgCRND8 mice following a single dose and following 6 days of *b.i.d.* drug administration. The increase in plasma A*β* observed at lower doses was more substantial in nontransgenic mice than what was observed in TgCRND8 mice following 6 days of *b.i.d.* administration which has been alluded to elsewhere [40].

In the cortex of nontransgenic mice, SCH 697466 dose-dependently lowered A*β*40 in a more linear fashion following 11 days of *q.d.* and 6 days of *b.i.d.* administration (Figure 5(a), main effect of dose: *F*(4,19) = 105.53 and *F*(4,21) = 101.04, resp., *P* < 0.0001). Cortical A*β* lowering was strikingly similar between the dosing paradigms with 50% lowering occurring around 30 mg/kg. The dose-responsive nature of cortical A*β* lowering observed here was similar to what was observed in TgCRND8 mice but did not plateau like what was observed in acutely rats. There was no evidence of an increase in A*β* in the cortex.

#### 3.5.2. Notch-Related Side Effects

Eleven days of *q.d.* administration of SCH 697466 at 30 and 100 mg/kg did not affect thymus weight, while 6 days of *b.i.d.* administration significantly decreased thymus weight by about 60% (Figure 5(b); main effect of dose: *F*(2,14) = 32.40, *P* < 0.0001) with significant weight reduction in the 100 mg/kg group, but not the 30 mg/kg group, compared to vehicle-treated mice (*P* < 0.0001). This is similar to what was observed in TgCRND8 mice treated for 6 days *b.i.d.* with 100 mg/kg SCH 697466.

Similarly, PAS staining in the ileum was not increased after 11 days of *q.d.* administration of SCH 697466 at 30 and 100 mg/kg, but 100 mg/kg SCH 697466 administered for 6 days *b.i.d.* markedly increased PAS staining in the ileum by about 350% (Figure 5(c); *F*(2,14) = 180.01, *P* < 0.0001; Figures 5(d)–5(g)). PAS staining in the 100 mg/kg 6 days *b.i.d.* nontransgenic group looked very similar to that observed in TgCRND8 mice following 100 mg/kg 6 days *b.i.d.* SCH 697466 (images not shown). PAS staining was not increased in the 30 mg/kg 6 days *b.i.d.* group.

Contrary to what as observed in the 6 day *b.i.d.* TgCRND8 study, there were no effects of SCH 697466 (up to 100 mg/kg) on body weight or survival in this nontransgenic study (data not shown).

#### 3.5.3. Notch-Related Biomarker Changes

HES-1 gene expression is directly affected by the Notch signaling pathway such that disrupted Notch activity leads to decreased HES-1 expression. Consequently, gene expression of two downstream targets of HES-1, mATH, and KLF4 should be increased following decreased HES-1 expression. As noted in the previous section, only the 100 mg/kg 6 day *b.i.d.* group showed Notch-related pathology.

**Figure 6:**
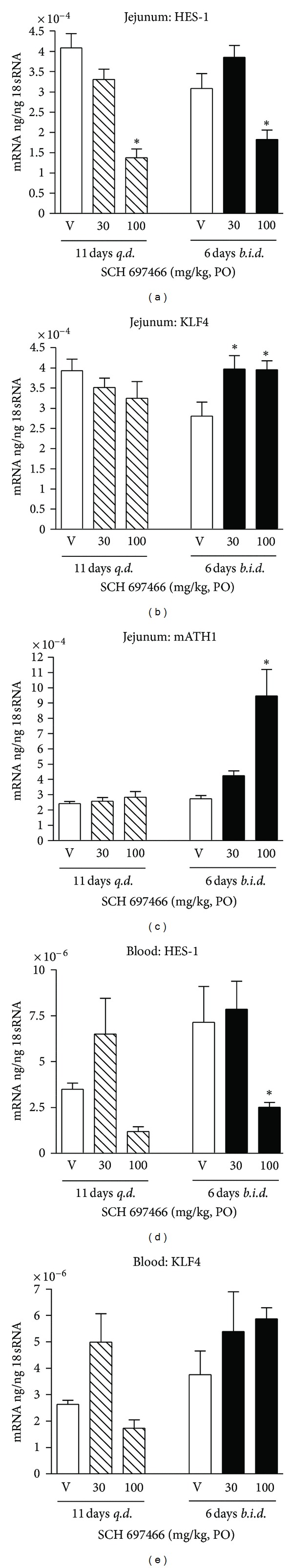
(a) HES-1, (b) KLF4, and (c) mATH gene expression in the jejunum and (d) HES-1 and (e) KLF4 gene expression in white blood cells following 11 days of *q.d.* administration (left side of graphs) or 6 days of *b.i.d.* administration (right side of graphs) of SCH 697466 in nontransgenic B6C3F1 mice. The final dose of SCH 697466 was given 3 hr prior to tissue harvesting. *P < 0.04 versus vehicle, Dunnett post hoc test.

Both 11 days of *q.d.* administration and 6 days of *b.i.d.* administration of SCH 697466 decreased HES-1 expression in the jejunum (Figure 6(a), main effect of dose: *F*(2,11) = 21.52 and *F*(2,13) = 14.71, resp., *P* < 0.0005), with significant decreases observed in both 100 mg/kg groups compared to vehicle-treated mice (*P* < 0.02). mATH1 and KLF4 expression was increased in the jejunum of mice treated for 6 days of *b.i.d.* SCH 697466 (Figures 6(b) and 6(c)); main effect of dose: *F*(2,13) = 7.19 and 4.51, *P* < 0.04) with increased expression in the 100 mg/kg group for mATH1 and 30 and 100 mg/kg group for KLF4 (*P* < 0.04). mATH1 and KLF expression was unchanged in the 11 day *q.d.* SCH 697466 groups (Figures 6(b) and 6(c)). Adipsin, another downstream target of HES-1, expression in the jejunum was very low in all groups (data not shown). 

In the blood, HES-1 expression was affected by treatment with SCH 697466 (Figure 6(d); main effect of dose: *F*(2,11) = 4.48 and *F*(2,13) = 6.86, *P* < 0.04) with decreased expression in the 100 mg/kg groups of both dosing paradigms (significant decrease for the 100 mg/kg *b.i.d.* group, *P* < 0.04). Although KLF4 expression was affected by SCH 697466 when administered for 11 days *q.d.* (Figure 6(e); main effect of dose: *F*(2,11) = 5.81, *P* < 0.02), none of the dosed groups differed from vehicle-treated mice. MATH1 and adipsin expression was not detected in the blood.

#### 3.5.4. Exposure

There was a dose-related increase (although in some cases it was more than dose-proportional) in the concentration of SCH 697466 in plasma and brain (3 hr posttreatment) in each dosing paradigm (Table 3). Overall, drug levels at corresponding doses were similar between the dosing paradigm groups and similar to what was observed acutely in rats, TgCRND8 mice, and nontransgenic mice and after 6 days of *b.i.d.* administration in TgCRND8 mice (except here we did not observe the very high brain exposure that was observed in the 100 mg/kg 6 days *b.i.d.* TgCRND8 group). Therefore, the Notch-related side effects and biomarker changes specifically observed in the 100 mg/kg 6 days *b.i.d.* group are not likely to be due to drug accumulation and higher exposure.

## 4. Discussion

Substantial evidence implicates A*β* peptides in the etiology of Alzheimer's disease. Given that A*β* is produced by cleavage of APP by *β*- and *γ*-secretase, *γ*-secretase inhibition is a promising disease modifying treatment for Alzheimer's disease. 

SCH 697466 [34] is a novel, potent, orally available *γ*-secretase inhibitor that reduced A*β* in plasma and brain of rats, preplaque TgCRND8 mice, and nontransgenic mice. The acute effects of SCH 697466 on A*β* levels were relatively similar between the rodent species and genotypes with about 50% lowering of A*β* in plasma, cortex, and CSF occurring between 10 and 30 mg/kg (or between ~0.4 and 3 *μ*M plasma concentration of SCH 697466) and near complete lowering of A*β* occurring at 100 mg/kg (or about 5–8 *μ*M plasma concentration). Therefore, as has been reported for other *γ*-secretase inhibitors, SCH 697466 was effective at reducing A*β* in vivo.

Although *γ*-secretase inhibitors reduce A*β* well in peripheral and central compartments, some *γ*-secretase inhibitors also produce mechanism-based side effects including a reduction in thymus size and an increase in intestinal goblet cell hyperplasia [5, 27–29]. Both of these effects are likely mediated by a disruption of Notch processing [30, 41, 42]. Based on our experience in rodents, these Notch-related side effects seem to be typically observed at doses that give continuous, near complete lowering A*β* in plasma for at least 3 days [5]. Alternatively, we and others have shown that “partial inhibition” doses of these same compounds provide reasonable lowering of A*β* (about >50% reduction) without Notch-related side effects [5, 18]. However, it is not clear how partial inhibition can best be applied in the management of Notch-related side effects, that is, is it preferable to aim for incomplete inhibition for a sustained period of time or complete inhibition for a short period of time? Therefore, we conducted additional subchronic studies with SCH 697466 to address this question as well as to assess the in vivo therapeutic window of this compound. 

As was observed acutely in TgCRND8 mice, subchronic *b.i.d. *administration of SCH 697466 dose-dependently lowered A*β* in plasma and cortex with about 50% inhibition occurring between 10 and 30 mg/kg (or between 2–10 *μ*M plasma exposure). SCH 697466 given at 100 mg/kg *b.i.d. *provided sustained and almost complete lowering of plasma A*β*, but also produced Notch-related side effects, including a reduction in thymus size and increased goblet cell hyperplasia. Alternatively, SCH 697466 given at 30 mg/kg *b.i.d. *to TgCRND8 mice provided sustained, although not complete, lowering of A*β* (48% reduction in cortex) without Notch-related side effects. Similar results were previously reported with another *γ*-secretase inhibitor, LY-411,575 [5]. Thus, although both the 30 and 100 mg/kg doses of SCH 697466 provided sustained lowering of A*β*, only the dose that also provided nearly complete lowering (100 mg/kg) produced Notch-related side effects, while the “partial” inhibition dose (30 mg/kg) did not. In other words, a short duration of action does not seem to be required to mitigate Notch-related side effects, just incomplete inhibition. 

Interestingly, although SCH 697466 had a reasonable in vitro separation between IC_50_s for A*β* lowering and Notch processing (~24-fold), this did not translate in vivo. After subchronic 6 day *b.i.d.* administration of SCH 697466, the ED_50_ for reducing brain A*β*40 was about 30 mg/kg and Notch-related side effects were observed at 100 mg/kg. Therefore, although there was a clear therapeutic window for SCH 697466 in vivo, it was small (~3-fold based on dose and ~5-fold based on plasma concentrations) and significantly less than the therapeutic window predicted by in vitro experiments. It is important to note that SCH 697466 was less potent at lowering the more amyloidogenic A*β*42 species especially in brain suggesting that the in vivo therapeutic index will likely be smaller if A*β*42 data were used instead. 

Given that the duration of action of SCH 697466 at 100 mg/kg is relatively long (>24 hr), both *b.i.d.* and *q.d.* administration would provide sustained and near complete lowering of A*β*. It was hypothesized, however, that *q.d.* administration of SCH 697466 might decrease the incidence of Notch-related side effects compared to those observed with *b.i.d.* administration while maintaining excellent lowering of A*β* (>80% reduction). Indeed, in a study with nontransgenic mice, although lowering of plasma and brain A*β* was very similar between the *b.i.d.* and *q.d.* dosing paradigms, the Notch-related side effects were strikingly different such that Notch-related pathology was only observed at 100 mg/kg when administered *b.i.d.* and not *q.d*. This did not seem to be due to large increases in exposure or drug accumulation in the *b.i.d.* group. Of note, although nontransgenic mice tolerated high doses of SCH 697466 better than TgCRND8 mice as measured by body weight loss and survival, both groups of mice showed similar reduction in thymus weight and increase in intestinal goblet cell hyperplasia at 100 mg/kg *b.i.d.* Thus, Notch-related side effects can be managed even with sustained and near complete lowering of A*β* by optimizing the frequency of dosing of *γ*-secretase inhibitors. However, it cannot be completely excluded that longer term administration of *γ*-secretase inhibitors even with an optimized frequency of dosing might eventually result in Notch-related side effects; further studies would be required to address this possibility. 

Recent data suggest that there may be additional options for mitigating Notch-related side effects with *γ*-secretase inhibition. A study by Das et al. [43] showed that treating transgenic mice with the *γ*-secretase inhibitor LY-411,575 for a short period of time during a key, early phase of amyloid accumulation resulted in long-lasting beneficial effects on brain A*β* without the need for continuous drug treatment. These data along with our own suggest that there may be ways to safely and effectively lower brain A*β* with *γ*-secretase inhibition. Further studies would be required to determine how useful these approaches would be in the clinic.

Based on our findings that Notch-related side effects can be managed with sustained but incomplete lowering of A*β*, or near complete lowering of A*β* but with more intermittent administration, it is possible that some of the “Notch-sparing” and “APP-selective” *γ*-secretase inhibitors reported in the literature may be managing Notch-related side effects by using these strategies instead of the molecules really being “Notch sparing” in vivo. For example, after 7 days of *b.i.d.* dosing of ELN 475516, an “APP-selective” *γ*-secretase inhibitor, there was no evidence of Notch-related toxicity as measured by thymus weight and goblet cell hyperplasia in the ileum and the doses and frequency of drug administration utilized provided sustained but incomplete (~74%) lowering of A*β* [10]. Perhaps if this compound was able to obtain near complete lowering of A*β* in a sustained fashion, Notch-related side effects may have indeed been observed.

It is important to note that in these studies, we were using reduction in thymus size and intestinal goblet cell hyperplasia as representative measures of “Notch-related side effects.” However, it is possible that there are other toxicities or effects related to *γ*-secretase inhibition and/or altered Notch signaling that we did not measure and that may not be mitigated or avoided with partial inhibition and/or intermittent dosing paradigms. Further, the translatability of these treatment approaches or mitigation strategies in rodents to humans is not clear.

Although some Notch-related side effects are reversible [5], it would be useful to have a clinically tractable biomarker to monitor potential effects of *γ*-secretase inhibitors on Notch processing in the clinic. HES-1 gene expression is directly affected by the Notch signaling pathway such that disrupted Notch activity leads to decreased HES-1 expression. Consequently, gene expression of two downstream targets of HES-1, mATH and KLF4, is increased following decreased HES-1 expression [32, 33]. In certain cases, the gene expression profiles of these Notch-related biomarkers paralleled the Notch-related pathology observed. For example, the 100 mg/kg dose of SCH 697466 when given *b.i.d.* induced Notch-related pathology and also decreased HES-1 in the jejunum and in the blood and increased mATH1 and KLF4 expression in the jejunum. The biomarker data did not completely align with the in vivo data though, for example, HES-1 gene expression was decreased in the jejunum of mice treated with 100 mg/kg *q.d.* when no pathology was observed and KLF4 gene expression was increased in mice treated with 30 mg/kg *b.i.d.* when no pathology was observed and when HES-1 expression (upstream of KLF4) was not decreased. It is also possible that the biomarker changes that were observed in the absence of pathology reflect relevant changes in Notch signaling that after a longer period of dosing would indeed result in observations of Notch-related pathology. In general, however, when clear Notch-related pathology was observed, changes in Notch-related biomarkers were simultaneously observed. This is in agreement with other reports using expression of these same genes as potential biomarkers for Notch-related pathology [10].

Interestingly and perhaps paradoxically in several studies an *increase* in plasma A*β* was observed at lower doses of SCH 697466 (e.g., 3 and 10 mg/kg). This increase in plasma A*β* or “A*β* rise” has been frequently observed with other *γ*-secretase inhibitors in vivo, including in humans, and occurs at low concentrations likely under conditions of partial enzyme occupancy [15, 40, 44, 45]. In addition, the A*β* rise was more prominent in nontransgenic mice than transgenic mice as has been supported elsewhere [40, 44]. Importantly, we never observed an increase in A*β* levels in the cortex with SCH 697466.

To summarize, SCH 697466 is a novel, potent *γ*-secretase inhibitor that is effective at reducing A*β* in plasma, CSF, and brain in rodents. SCH 697466 has a small, but distinct, in vivo therapeutic index between A*β* lowering and Notch-related side effects in the ileum and thymus in mice. A partial inhibition dose of SCH 697466 provided reasonable reduction of A*β* (~50%) that was sustained over 24 hr when given *b.i.d.* without evidence of Notch-related side effects. Although the reasons are not entirely clear, *q.d.* dosing provided another way to maintain excellent, continuous inhibition of A*β* (>80%) with SCH 697466, but without Notch-related side effects. Therefore, by utilizing partial, but sustained, inhibition doses or optimizing the frequency of administration, it may be possible to obtain robust and sustained lowering of A*β* without Notch-related side effects using *γ*-secretase inhibitors. Finally, HES-1 expression in blood may be a useful Notch-related biomarker in the clinic. 

## Figures and Tables

**Table 1:** Summary of in vivo studies conducted with SCH 697466.

Group	Species	Duration of dosing	Total # of doses	Post-treatment time*	*n*/group
Acute Rat	Rat	single dose	1	3 hr	7–8
Acute TgCRND8	Mouse	single dose	1	3 or 6 hr	4–6
Acute Non-transgenic	Mouse	single dose	1	3 hr	7
Time course TgCRND8	Mouse	single dose	1	2, 4, 6, 12, 18 or 24 hr	4-5
6 days *b.i.d.* TgCRND8	Mouse	6 days *b*.*i*.*d* ^†^	11	3 hr	4–9
11 days *q.d.* Non-transgenic	Mouse	11 days *q.d. *	11	3 hr	4-5
6 days *b.i.d.* Non-transgenic	Mouse	6 days *b*.*i*.*d* ^†^	11	3 hr	4–7

*Time in between administration of the last (or only) dose and collecting tissues following euthanasia.

^†^Five full days of dosing with the final dose given the morning of day 6.

**Table 2:** Concentration of SCH 697466 (*μ*M) in plasma and brain for various acute studies in rats, TgCRND8 mice and non-transgenic mice.

Dose (mg/kg)	Rat		TgCRND8 mouse				Nontransgenic mouse	
	3 hr post-Treatment time		3 hr post-Treatment time		6 hr post-treatment time		3 hr post-Treatment time	
	Plasma	Brain	Plasma	Brain	Plasma	Brain	Plasma	Brain
3	nd	nd	nd	nd	0.05 ± 0.01	0.008 ± 0.002	0.03 ± 0.003	BLOQ
10	0.60 ± 0.07	0.13 ± 0.01	0.68 ± 0.09	0.04 ± 0.004	0.36 ± 0.02	0.06 ± 0.01	0.35 ± 0.05	0.01 ± 0.002
30	2.34 ± 0.16	1.33 ± 0.08	3.13 ± 0.26	0.96 ± 0.22	4.22 ± 1.27	0.36 ± 0.05	1.91 ± 0.12	0.18 ± 0.02
100	4.60 ± 0.39	4.49 ± 0.50	7.79 ± 1.33	9.71 ± 2.95	3.76 ± 1.19	1.99 ± 0.26	6.90 ± 0.81	3.04 ± 0.36

nd: Not done; BLOQ: below the limit of quantification (0.003 *μ*M).

**Table 3:** Concentration of SCH 697466 (*μ*M) in plasma and brain for various sub-chronic studies in TgCRND8 and non-transgenic mice.

Dose (mg/kg)	TgCRND8 mouse		Nontransgenic mouse			
	6 days *b.i.d. *		11 days *q.d. *		6 days *b.i.d. *	
	Plasma	Brain	Plasma	Brain	Plasma	Brain
3	0.02 ± 0.003	0.003 ± 0.003	0.03 ± 0.01	BLOQ	0.06 ± 0.04	BLOQ
10	0.47 ± 0.06	0.05 ± 0.01	0.43 ± 0.06	0.01 ± 0.0002	0.35 ± 0.05	BLOQ
30	2.64 ± 0.29	0.32 ± 0.05	2.99 ± 0.22	0.20 ± 0.03	2.03 ± 0.56	0.10 ± 0.02
100	ns	14.96 ± 5.44	8.10 ± 0.84	2.63 ± 0.52	10.21 ± 0.68	3.15 ± 0.54

ns: No samples; BLOQ: below the limit of quantification (0.003 *μ*M).
